# Supplementary material for: Human Macrophages Polarized by Interaction with Apoptotic Cells Produce Fibrosis-Associated Mediators and Enhance Pro-Fibrotic Activity of Dermal Fibroblasts In Vitro
Source: Cells. 2023 Jul 25;12(15):1928. doi: 10.3390/cells12151928 (PMC10417661; doi:10.3390/cells12151928)
Supplement: Supplementary file 1 [file cells-12-01928-s001.zip › cells-2507426-supplementary.pdf]

cells

MDPI

Supplementary Materials

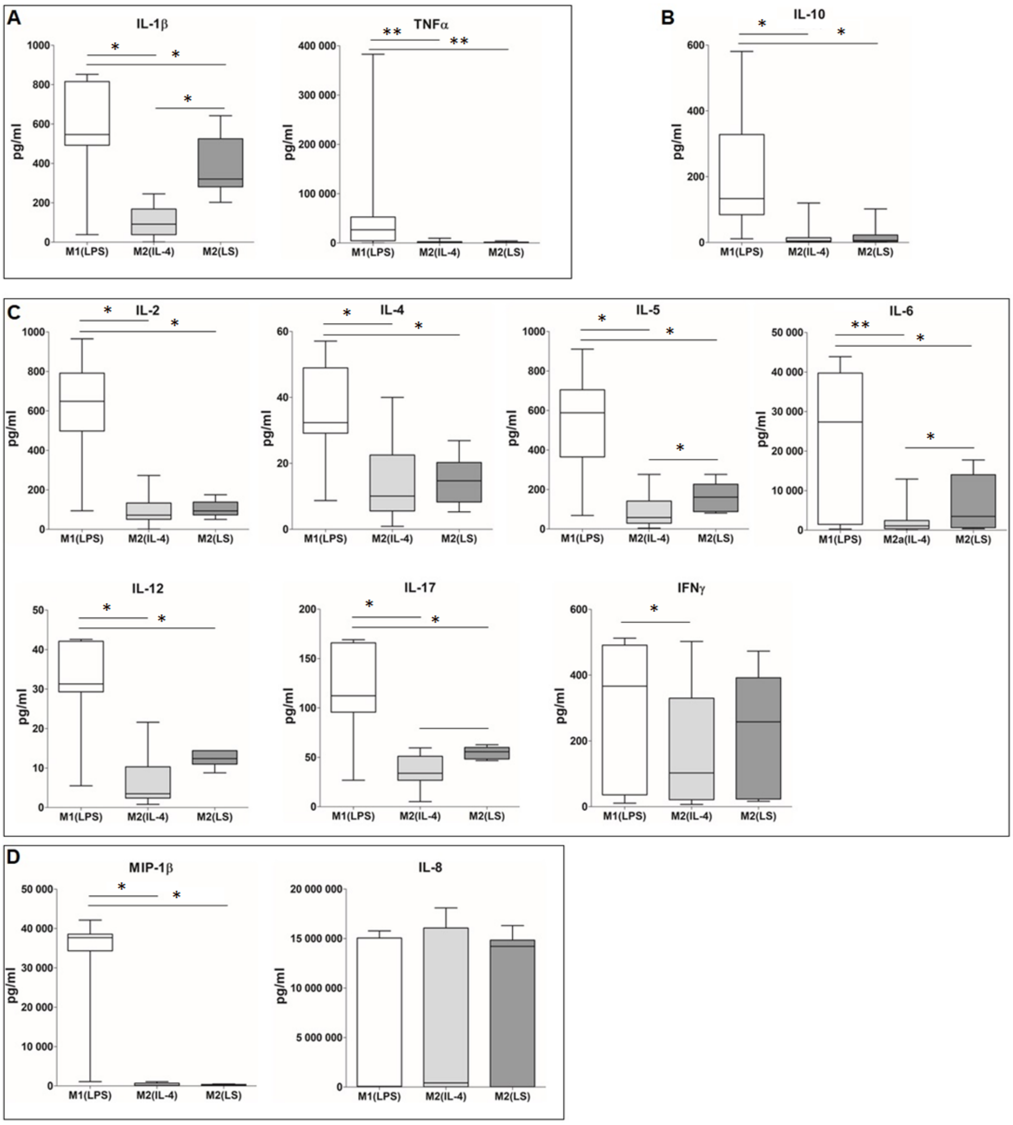

Figure S1. Cytokines and chemokines produced by M1(LPS), M2(IL-4), and M2(LS) macrophages. Data are presented as median, interquartile range, minimum and maximum, n=6-16. * significant at <0.05, ** significant at <0.01. (**A**) Pro-inflammatory cytokine (IL-1, TNFα) production (n=6-16). (**B**) Anti-inflammatory (IL-10) cytokine production (n=16). (**C**) Immunoregulatory cytokine (IL-2, -4, -5, -6, -12, -17, IFN-γ) production (n=6-16). (**D**) Chemokine (MIP-1β, IL-8) production (n=6-16).
